# Direct transmission of severe fever with thrombocytopenia syndrome virus from farm-raised fur animals to workers in Weihai, China

**DOI:** 10.1186/s12985-024-02387-x

**Published:** 2024-05-17

**Authors:** Jizhao Li, Chunping Wang, Xiang Li, Guoying Zhang, Shunzeng Sun, Zhefeng Wang, Jian Zhao, Linqing Xiu, Nianchen Jiang, Huajiang Zhang, Zhenghui Yang, Jinbo Zhang

**Affiliations:** 1 Department of Infectious Disease Control, Weihai Center for Disease Control and Prevention, 5A-1 Nanyuan Rd, Huancui District, Weihai, Shandong Province P.R. China; 2 Microbiology Laboratory Department, Weihai Center for Disease Control and Prevention, Weihai, Shandong Province P.R. China; 3 Department of Infectious Disease Control, Wendeng Center for Disease Control and Prevention, Weihai, Shandong Province P.R. China; 4 Weihai Health Commission, Weihai, Shandong Province P.R. China

**Keywords:** Severe fever with thrombocytopenia syndrome (SFTS), Fur animals, SFTSV whole-genome, Phylogenetic analyses

## Abstract

**Background:**

Severe fever with thrombocytopenia syndrome (SFTS) is an emerging infectious disease. SFTS virus (SFTSV) is transmitted by tick bites and contact with the blood or body fluids of SFTS patients. Animal-to-human transmission of SFTS has been reported in Japan, but not in China. In this study, the possible transmission route of two patients who fed and cared for farm-raised fur animals in a mink farm was explored.

**Method:**

An epidemiological investigation and a genetic analysis of patients, animals and working environment were carried out.

**Results:**

It was found that two patients had not been bitten by ticks and had no contact with patients infected with SFTS virus, but both of them had skinned the dying animals. 54.55% (12/22) of the farm workers were positive for SFTS virus antibody. By analyzing the large, medium and small segments sequences, the viral sequences from the two patients, animals and environments showed 99.9% homology.

**Conclusion:**

It is suspected that the two patients may be directly infected by farm-raised animals, and that the virus may have been transmitted by aerosols when skinning dying animals. Transmission by direct blood contacts or animal bites cannot be ignored.

## Introduction

Severe fever with thrombocytopenia syndrome (SFTS) is an emerging infectious disease caused by the Dabie bandavirus, which was first isolated from a man in rural areas of central China by Yu et al. in 2009 [1], and the disease was subsequently reported in 27 Provinces in China. The disease was later reported in other countries, including Korea, Japan and Vietnam [2–4], and a disease similar to SFTS caused by the Heartland virus was reported in the United States in 2012 [5].

The major clinical manifestations of SFTS include acute fever, thrombocytopenia, leukocytopenia, gastrointestinal symptoms (such as diarrhea, nausea and vomiting), and neurological symptoms (such as drowsiness, lethargy and slow response), and other less specific clinical manifestations. In severe cases, SFTS may lead to multiple organ failure or even death. The case fatality rate of SFTS is about 2.5–30% in China [6] and approximately 16% in Japan [7]. Currently there is no specific remedy and vaccine against SFTS; Ribavirin, Favipiravir and supportive treatment, including treatment with intravenous immunoglobulin, plasma exchange and corticosteroid have been reported to be beneficial for SFTS [8].

SFTS is mainly considered a vector-borne infectious disease and *Haemaphysalis longicornis* is thought to be the primary vector of SFTS virus (SFTSV) [9]. *Haemaphysalis longicornis* has a wide range of hosts, including domesticated and wild animals. Two studies conducted in Shandong Province showed that SFTSV-specific antibodies were detected in 69.5% of sheep, 60.5% of cattle, 47.4% of chickens, 37.9% of dogs, 8.4% of minks and 3.1% of pigs, and the sequences of SFTSVs from these domesticated animals were highly similar (> 95% homology) to human isolates from the region [10, 11]. Human-to-human transmission of SFTS has been confirmed and the interest in animal-to-human disease transmission has increased after direct transmission of SFTSV from cats to humans [7, 12, 13] and dogs to human [14] were firs reported in Japan, but there are no reports of animal-to-human transmission of SFTS in China. Therefor, it is difficult to propose new prevention and control strategies from the aspects of managing animal infectious sources and cutting off transmission routes.

In this study, a cluster of SFTS cases was reported, in which two patients worked on the same mink farm in Shandong Province, China. At the same time, some foxes and minks died without definite causes. Additionally, the epidemiological investigation of two patients was analyzed and laboratory testing was performed on the workers, foxes and environments of the mink farm.

## Methods

### Subjects

A cluster of two SFTS-liked patients was identified in Wendeng County, Shandong Province on October 21, 2023. A 60-year-old woman, who presented symptoms of nausea and vomiting on October 17, was admitted to Hospital A in a coma on October 19, and subsequently died on October 25. The other case in the cluster was her colleague on the same mink farm who developed a fever on October 15. Anyone who lived, worked, or physically contacted with the two patients from October 15 to October 24 were defined as a close contact. 24 close contacts were examined by the investigators.

### Epidemiological investigation

An epidemiological study was conducted on the two patients to investigate possible transmission of the cluster. Data were collected in-person by well-trained interviewers to obtain the exposure history, and medical records were obtained from their visiting hospital. The basic situation of this mink farm was investigated through the manager. In order to obtain the infection status of close contacts, the venous blood (5 mL) was collected from 22 workers of the mink farm and 2 family members who cared for the patients. In addition, sera from two foxes in the farm and environmental samples from the slaughter floor and enclosure zone were also collected.

### Tick collection

Ticks were collected on the mink farm and in the fields near the patients’ home from October 24 to October 31. Ticks were collected with 1 m^2^ white flannel flag by flagging over the plant-life. Farm-raised fur animals were also checked for ticks on their bodies.

### Laboratory analysis

Venous blood (5 mL) samples were collected from close contacts and two foxes. Environmental samples were collected with yocon non-inactivated virus sampling kit (MT0901-3-3.5) for nucleic acid testing of SFTSV. The viral RNA was extracted from serum samples or environmental smear samples using the Automatic nucleic acid extraction instrument (Jiangsu Bioperfectus Technologies Co, Ltd).Then RNA was amplified using the SFTSV Real-time PCR Kit (Jiangsu Bioperfectus Technologies Co., Ltd). Serum samples from patients, foxes and close contacts were tested using SFTSV IgM antibody ELISA detection kit and SFTSV IgG antibody ELISA detection kit(Zhongshan Bio-tech Co., Ltd).

Samples with positive Realtime PCR results and cycle threshold value (CT) < 32 were selected for whole genome sequencing. The viral RNA was extracted using the QIAamp Viral RNA Mini Kit (Qiagen,52,904). The RNA was reverse transcribed and amplified using the SFTSV Multiplex amplicon whole-genome enrichment reagents(Shanghai BioGerm Medical Technology Co., Ltd). The PCR products were purified using AMPure XP magnetic beads (Beckman Coulter, Brea, CA, USA). Library construction was performed using the Nextera XT DNA Library Preparation Kit and Nextera XT Index Kit (Illumina, San Diego, CA, USA). Whole genome sequencing was performed using Illumina Nextseq2000 sequencer. The sequencing read length was set to 2 × 151.

SFTSV genome aligment and assembly were performed using CLC Genomics Workbench version22.0. QC analysis, trimming and mapping were performed to obtain consensus sequences of L, M, S segments. HB29 (NC_018136.1, NC_018138.1, NC_018137.1 for L, M, S segments) was used as the reference. The sequence alignment was computed using the MAFFT software(version 7.037b). The Bioedit software (version 7.2.5) was used to calculate S, M, and L segments Sequence Identity Matrix and Sequence differences count Matrix. The phylogenetic tree was constructed using MEGA (version 11). The phylogenetic tree was constructed with Maximum Likelihood (ML) method, and the Bootstrap value was set to 1000 times to evaluate the reliability of the phylogenetic tree. Sequences from the Heartland virus were used as outgroups.

## Results

### Clustered cases

Patient 1 was a 60-year-old woman, who worked on a mink farm in Wendeng County. On October 17, she developed vomiting, dizzy and fatigue and self-medicated with Montmorillonite powder at home. Her symptoms worsened and she was diagnosed with “unconsciousness to be examined” in Hospital A on October 19. Laboratory results revealed white blood cell (WBC) and platelet counts of 2.47 × 10^9^/L and 61 × 10^9^/L, respectively. The creatine kinase, lactic dehydrogenase, AST, ALT, creatinine, and urine protein were 1873 U/L, 1427 U/L, 790 U/L, 169 U/L, 303 U/L and 3+, respectively (Table 1). The test results showed that possible organ impairment in her heart, liver and kidneys and she was transferred to the intensive care unit. Based on the prevalence of SFTS in Wendeng County and the laboratory results of the patient, the doctor suspected that the patient was infected with SFTSV. On October 21, the patient was tested for SFTSV, and the SFTSV copy number was 3.7 × 10^4^ TCID50/mL. On the same day, her colleague was also diagnosed with SFTS. Subsequently, her platelet counts declined below the admission levels (35 × 10^9^/L). Considering her poor condition, her relatives decided to bring her home on October 25 after being diagnosed with severe encephalitis and SFTS, and she died the same day.

Patient 2 was a colleague of patient 1 but they did not live in the same village. She became feverish on October 15 accompanied by chills, fatigue and soreness. The patient had a maximum body temperature of 38℃ and self-administered ibuprofen granules and Lianhua Qingwen Granules at home. On October 18, she went to the township hospital where she was diagnosed with “granulocytopenia”. On October 20, she was transferred to Hospital A where she was diagnosed with “ fever of unknown origin”. Laboratory results revealed white blood cell(WBC) and platelet counts of 1.98 × 10^9^/L and 96 × 10^9^/L respectively. The creatine kinase, lactic dehydrogenase, AST, ALT, creatinine, and urine protein were 316 U/L, 366 U/L, 62 U/L, 37 U/L, 82 U/L and negative, respectively on October 21. She was diagnosed with SFTS on the same day as Patient 1, and the SFTSV copy number was 1.2 × 10^4^ TCID50/mL (Table 1). She received Ribavirin and supportive treatment, after which her condition was getting better and better. She was discharged on November 6.

**Table 1:** Serum laboratory information of the 2 patients with SFTS

	Patient1		Patient2		
Parameter	admission	discharge	admission	discharge	Reference Range
WBC (10^9^/L)	2.47	5.83	1.98	3.68	3.5–9.5
PLT (10^9^/L)	61	35	96	252	100–350
CK(U/L)	1873	1578	316	39	20–170
CK-MB(U/L)	66.6	85.5	20.3	1102	0–24
LDH(U/L)	1427	3843	366	496	120–250
ALP(U/L)	144	150	59	153	40–130
ALT(U/L)	169	82	37	94	9–50
AST(U/L)	790	584	62	50	15–40
Ur(mmol/L)	24.7	19.7	5.5	ND	3.6–9.5
Cr(µmol/L)	303	311	82	ND	57–111
Upro	3	ND	0	ND	-
CRP (mg/L)	ND	ND	3.2	ND	0–3

WBC, white blood cells; PLT, platelet; CK, creatine kinase; CK-MB, creatine kinase myocardial isoenzyme; LDH, lactate dehydrogenase; ALP, alkaline phosphatase; ALT, alanine aminotransferase; AST, aspartate aminotransferase; Ur, Urea; Cr, creatinine; Upro, urine protein; CRP, C-reactive protein

### Epidemiological investigations

According to the Technical Specifications for Monitoring in China, clustered cases are defined as 2 or more cases occurring in the same location within 2 weeks. The two patients worked on the same farm and were diagnosed with SFTS on the same day, so they were defined as clustered cases, and epidemiological investigations were conducted to find the potential transmission route of the two patients. Epidemiological investigation showed that the two patients had no history of tick bites or tick-bite scars prior to the onset of SFTS. Their regular job on the mink farm was to feed farm animals with dedicated feeding carts and sometimes they also skinned the dying animals. They might be scratched by animals or come into direct contact with animal blood during work, but they had poor awareness of self-protection during work. The farm was then inspected.

**Fig. 1:**
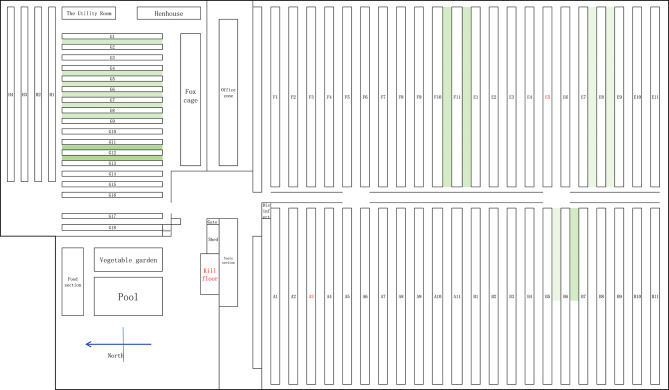
The plan of the mink farm. A and B zones were for raising minks; E, F and G zones were for raising foxes; H zone was for raising raccoon dogs. The working zone of Patient 1 was A3, and that of Patient 2 was E5. The green areas were grassland

The mink farm was located 1 km northeast of Houjia Village, Houjia Town, Wendeng County, where more than 6000 minks, 10,000 foxes and 500 raccoon dogs were raised. Various animals were raised in different zones, and the entire field was divided into 6 zones. A, B, E and F zones were located on the south side of the gate, with 11 rows in each zone. Of these, A and B zones were for raising minks and the E and F zones were for raising foxes; G zone was located on the north side of the gate, with a total of 18 rows, for raising foxes; H zone was located on the north side of G zone, with a total of 4 rows, for raising raccoon dogs (Fig. 1). There were more than 20 employees on the farm, each responsible for raising, cleaning and disinfecting animals in a certain zone, and for skinning dying animals.

**Fig. 2:**
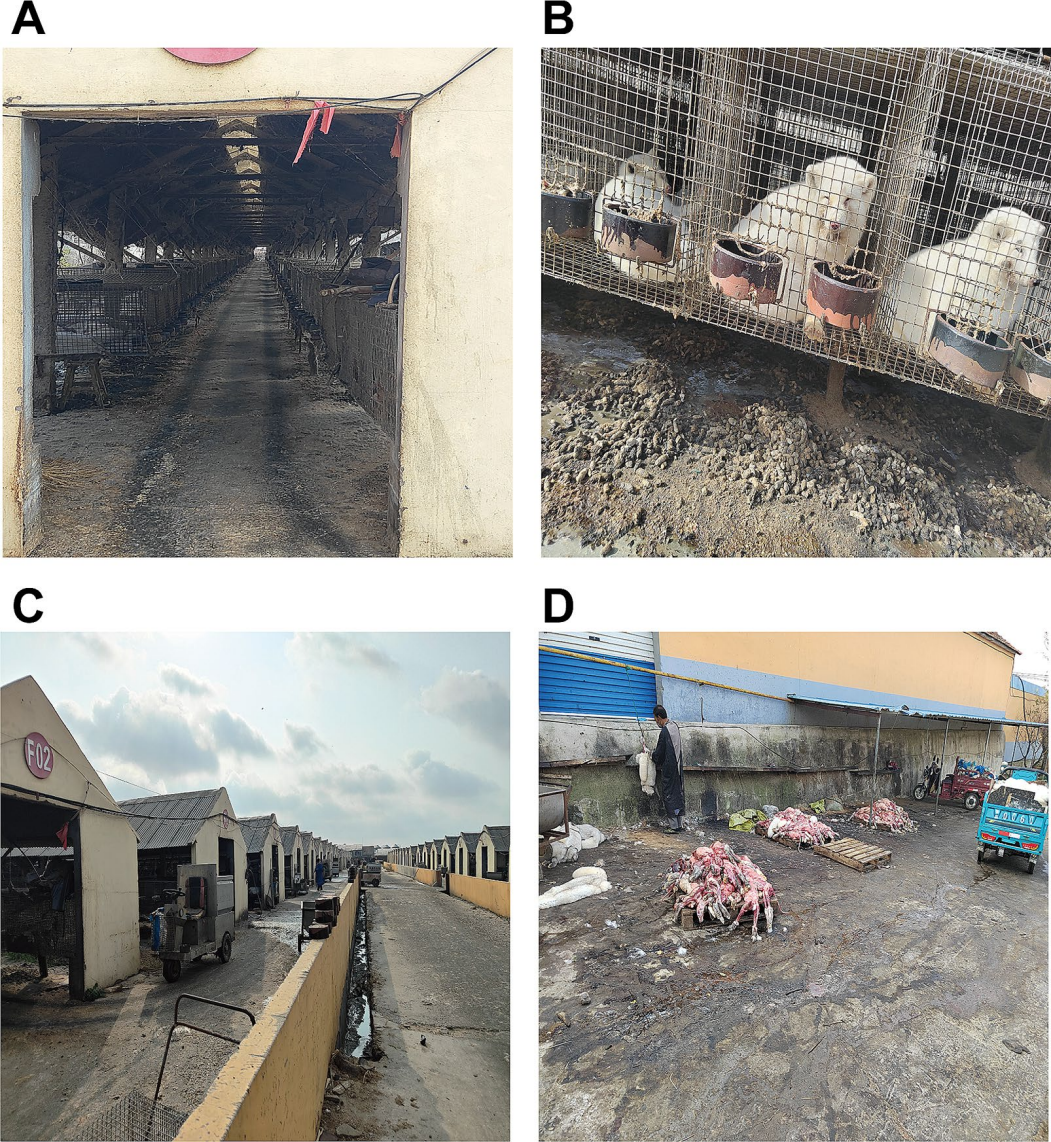
cage house (**A**, **B**); automatic feeding cart (**C**); the slaughter floor (**D**)

The working zone of Patient 1 was A3, and that of Patient 2 was E5. Workers fed the animals automatically on electric feeding carts, with minimal contact with animals. During the process of skinning animals, there might be contact with the animals, leading to the possibility of being scratched or bitten. 22 workers who had worked on the mink farm for many years were investigated. 17 of whom had been scratched or bitten by foxes or minks while skinning the dying animals. The skinning area was an open space in front of the gate outside the venue, for skinning of animals throughout the venue (Fig. 2). The workers wore protective equipment such as aprons, gloves, and masks when skinning animals, but the protection was not standardized, posing a risk of infection.

### Tick collection

Previous tick monitoring showed the distribution of *Haemaphysalis Longicornis* in Houjia Town. An attempt was made to collect the ticks on the mink farm and in the fields near the patients’ home from October 24 to October 31, but no tick was captured. No ticks were found on the animal bodies either.

### Virus detection

Realtime PCR was performed on 22 workers, the results of which showed that none of them were positive for SFTSV, but 54.55% (12/22) of them were positive for SFTSV antibody. Except for one worker tested positive for SFTSV-specific IgM, all others were tested positive for SFTSV-specific IgG, which was considered a prior infection. 2 family members tested negative for SFTSV. Two foxes were positive for SFTSV but negative for IgM and IgG, which was considered a recent infection. 5 animal skinning environmental samples were taken for Realtime PCR, the results of which showed that all environmental samples were SFTSV-positive, while another 7 environmental samples from A, B, F and F zones were SFTSV-negative (Table 2).

**Table 2:** Realtime PCR test results of patients, foxes and environments

Realtime PCR	site	sampling time	CT
Patient1	/	20-Oct	22.5
Patient2	/	21-Oct	24.9
Fox1	/	24-Oct	16.7
Fox2	/	24-Oct	12.9
E1	skinning tool	24-Oct	26.6
E2	hook	24-Oct	24.3
E3	ground	24-Oct	22.3
E4	ground	24-Oct	34.1
E5	dustbin	24-Oct	21.2
E6	A1-A5	5-Nov	-
E7	A6-A11	5-Nov	-
E8	B1-B5	5-Nov	-
E9	B6-B11	5-Nov	-
E10	E1-E5	5-Nov	-
E11	E6-E11	5-Nov	-
E12	F1-F5	5-Nov	-
E13	F6-F11	5-Nov	-

CT, cycle threshold value

### Phylogenetic analyses

**Fig. 3:**
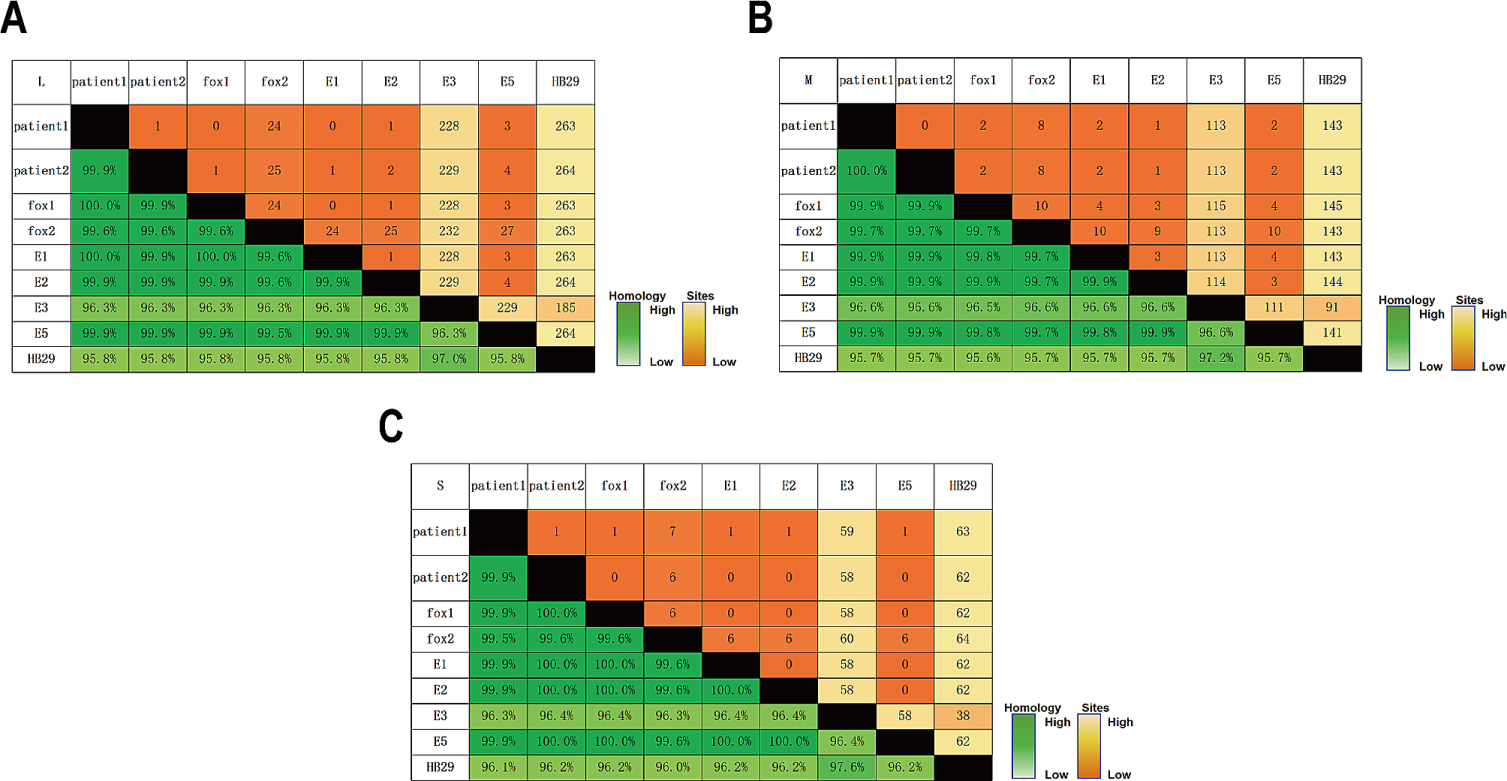
The percentage homology and pairwise distance of 8 SFTSV sequences in this outbreak for L(**A**), M(**B**), S(**C**) segments. HB29 (NC_018136.1,NC_018138.1,NC_018137.1 for L, M,S segments) was used as the reference

A total of 8 SFTSV whole genome sequence samples were obtained, including 2 patient samples, 2 fox samples and 4 environmental samples from mink farm. By analyzing the L, M, and S segments sequences of 8 samples, it could be found that 6 out of 8 samples, including Patient1, Patient2, fox1, E1, E2 and E5, showed high homology. Pairwise distance analyses showed that the single nucleotide mutations diversities between these 6 sequences were no more than 4 sites (range: 0~4 SNPs). The homology of nucleotides between these 6 samples was up to 99.9%. In addition, the phylogenetic analysis of L, M and S segments also showed that all 6 samples belonged to the same branch. These results indicated that the SFTSV in the 6 samples belonged to the same source (Fig. 3).

**Fig. 4:**
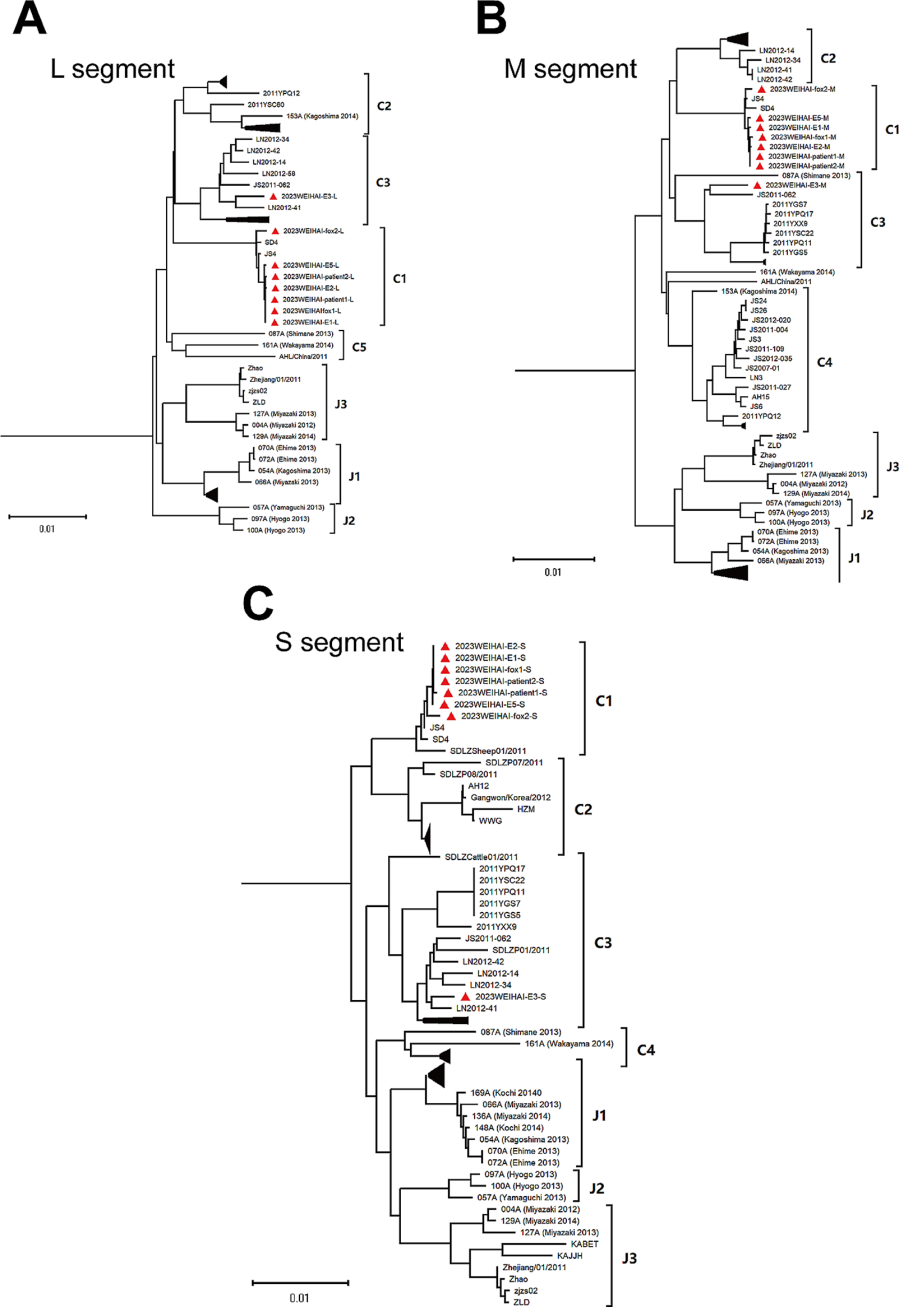
The phylogenetic trees of the SFTSV genome for the L(**A**), M(**B**), S(**C**) segments. The taxon names marked with red triangles are the sequences of this outbreak. Sequences from the Heartland virus used as outgroups were not shown in the figures Some subtrees that do not affect the typing was compressed with black triangles

Due to the lack of a unified classification method for SFTSV gene subtypes at present, these 8 samples were classified according to Tomoki Yoshikawa classification method [15]. Phylogenetic analysis showed that 6 sequences, including Patient1, Patient2, fox1, E1, E2 and E5, belonged to C1 gene subtypes and formed a cluster in the phylogenetic tree. The sequence of fox2 also belonged to C1 gene subtypes, but there were significant differences in mutation sites compared to the six samples. Therefor, they did not belong to the same cluster. These 7 sequences belonged to the same gene subtype as those uploaded from Shandong in 2010 (HM802202, HM802203, HM802204) and Jiangsu in 2010 (HQ141604, HQ141605, HQ141606). According to the results of the gene typing, the sequence of E3 belonged to C3 gene subtypes. The L and S segments of E3 belonged to the same branch as the sequences uploaded from Liaoning Province in 2012 (KF887443, KF887433) (Fig. 4).

### Risk factors for transmission of SFTSV

To assess the risk factors for transmission of SFTSV, a case-control study was conducted on 22 workers. 12 workers positive for SFTSV antibody were categorized into the cases group, and 10 workers negative for SFTSV antibody were categorized into the control group. As shown in Table 3, direct contact with animal blood and animal bites were found not to be associated with SFTS.

**Table 3:** Risk factors assessment for transmission of SFTSV

	Overall	case	Odds ratio(95%CI)	*P* ^a^
Animal blood				0.07
Exposed	15	6	0.11(0.01–1.17)	
Unexposed	7	6		
Animal bite				0.38
Exposed	15	7	0.35(0.05–2.41)	
Unexposed	7	5		

^a^ Fisher’s precision probability test

## Discussion

SFTS is an infectious disease of global concern, and China has the highest reported incidence of SFTS. Since the emergence of SFTS in 2009, tick bites have been identified to be the most likely route of transmission, and the farmers engaged in agricultural activities in the hilly areas have the highest risk of infection [16, 17]. SFTSV can also be transmitted human-to-human through contact with the blood, secretions or mucous of SFTS patients [18, 19], however, some cases of SFTS have been demonstrated direct cat-to-human or dog-to-human transmission in Japan [7, 12–14]. But no studies have reported animal-to-human transmission in China.

The fur animal breeding industry has developed rapidly in China, and the number of minks and foxes skinned in Shandong Province has ranked first in recent years. The fur animal breeding industry in Wendeng County has reached a considerable scale, especially in Houjia Town where the farm we studied is located. Mink, fox and raccoon dog are the main fur animals raised with high economic value. The virus monitoring of these fur animals mainly involves canine distemper virus, parvovirus, Aleutian virus and common bacteria such as Pseudomonas aeruginosa, Salmonella and Pasteurella. The electronic databases were searched for articles on fur animals with the search words and terms in English and Chinese: (“SFTS” or “SFTSV” or “Severe fever with thrombocytopenia syndrome” or “Severe fever with thrombocytopenia syndrome virus”) and (“fox” or “mink” or “raccoon dog”). Finally, 3 studies [11, 20, 21] were identified, only one of which was conducted on Chinese minks. SFTSV antibodies were found in 23% (12/53) of Japanese raccoon dogs [20], none in Korean raccoon dogs [21] and in 8.4% (15/178) of Chinese minks [11]. The three studies indicated that fur animals may be highly seropositive to SFTSV.

In this study, the two patients were successively infected with SFTSV in October. This cluster of cases had caught our attention, and an epidemiological investigation was conducted immediately on the two patients to identify the source of infection. The results of epidemiological investigations ruled out tick bites or human-to-human transmission as the two patients reported no tick bite and no contact with patients infected with SFTSV. No ticks were captured on the mink farm and in the fields near the patients’ home. Considering that the two patients had skinned dying animals prior to the onset of SFTS, 5 samples were taken from the environment where the animals were skinned, and two serum samples were collected from two dying foxes. Realtime PCR revealed that all samples tested were positive for SFTSV RNA, and another 7 environmental samples from A, B, E and F zones were negative for SFTSV. Results of animal and environmental studies indicated that infected animals contaminated the environment when skinned.

In this study, 54.55% of 22 workers were SFTSV antibody positive, and 77.27% were confirmed to have had direct contact with blood when skinning and have been bitten by the animals they raised, but none of them had been bitten by ticks. Case-control study showed that direct contact with animal blood and animal bites were found not to be associated with SFTS. Considering the results of environmental samples from the slaughter floor and the poor self-protection awareness, the mode of transmission was hypothesized to be via aerosol, however, direct contact with animal blood and animal bites cannot be excluded.

Genome sequencing was conducted to further prove that the patients were infected with SFTSV, when skinning SFTSV-infected animals. Phylogenetic analysis showed that 6 sequences, including Patient1, Patient2, fox1, E1, E2 and E5, belonged to C1 gene subtypes, and the homology of nucleotides between these 6 samples was up to 99.9%. The sequence of fox 2 also belonged to C1 gene subtype with significant differences in mutation sites compared to the six samples. High homology of the viruses isolated from the patients, fox and environments provided genetic evidence for the epidemic findings on the transmission of SFTS. On the other hand, it suggested that animals had a high seropositivity for SFTSV.

Ticks have been confirmed to be the main vector of transmission for SFTSV, however, an increasing number of SFTS cases have no direct or indirect contact with ticks or patients infected with SFTS [23–26], and their routes of transmission are unknown. A study [27] conducted by Yang et al. reported that some SFTS patients had a history of mink bite in Shandong Province. In this study, a direct farm-raised fur-animal-to-human transmission was demonstrated for the following reasons: two patients had no direct or indirect contact with ticks, and no ticks were captured on the mink farm and in the fields near the patients’ home; the complete nucleotide sequence of SFTSV from the two patients, fox and 3 environmental samples showed 100% homology; the seroprevalence of SFTSV in workers on the mink farm was 54.55%, and all of them had skinned dying animals; a large number of farm-raised animals became sick or died of unknown causes during SFTS epidemic period.

There are some limitations to our study. Firstly, the animal sample sizes and the specimens in case-control study were relatively small. Secondly, no mode of animal-to-human transmission was found. Thirdly, virus could not be isolated due to laboratory constraints. Lastly, no ticks were captured, and no virus sequence of ticks could be isolated.

## Conclusions

It is inferred that the two patients are infected with SFTSV through skinning SFTSV-infected animals. If a patient engaged in fur animal farming develops SFTS-like symptoms during SFTS epidemic period, SFTS should be considered first. Further, fur animals should be regularly monitored for SFTSV infection, and attention should be paid to occupational exposure of farm workers when handling animals. Strict self-protection measures (e.g., protective clothing and masks, rubber gloves and disinfection equipment) need to be adopted to reduce the risk of exposure to the virus.

## Data Availability

The datasets generated and/or analysed during the current study are available from the corresponding author on reasonable request.
